# Evaluating the Impact of a Digital Detox Intervention Among Medics in Coventry and Warwickshire Partnership NHS Trust

**DOI:** 10.1192/bjo.2025.10208

**Published:** 2025-06-20

**Authors:** Onyedikachi Onyeaso, Nadia Saleem, Rupinder Kaler

**Affiliations:** Coventry and Warwickshire Partnership NHS Trust (CWPT), Coventry, United Kingdom

## Abstract

**Aims:** The excessive use of digital devices and social media has been associated with stress, poor focus, and a decline in overall well-being. Healthcare professionals, often working in high-pressure environments, are particularly susceptible to these effects due to their reliance on digital tools for work and personal purposes. This study aimed to assess the feasibility, adherence, and outcomes of a two-week digital detox intervention among medical professionals, focusing on its impact on digital device usage, stress levels, productivity, and overall well-being.

**Methods:** Thirty medics were invited to participate in a structured digital detox programme. Participants were given the option to either completely abstain from using digital devices or reduce their overall usage, with a particular focus on limiting social media engagement. Surveys were conducted at three stages: before the intervention to capture baseline device usage and social media habits, during the intervention to assess adherence, and after the detox to evaluate outcomes. Participants were encouraged to document their experiences through diaries or video reflections. The collected data included quantitative measures (e.g., screen time, adherence rates) and qualitative feedback on participants’ challenges and perceived benefits.

**Results:** Of the 30 invited participants, 24 (80%) agreed to participate in the digital detox, with 20 (83%) completing the two-week intervention. Pre-detox surveys revealed that participants spent an average of 5.5 hours daily on digital devices, with 40–50% of that time dedicated to social media. Post-intervention findings highlighted significant improvements, with 60% of participants reporting enhanced focus and productivity, 50% experiencing reduced stress levels, 40% noting improved sleep quality, and 30% engaging more in offline activities, such as hobbies and personal relationships.

However, challenges were reported, particularly during the initial stages, with 50% of participants experiencing restlessness or boredom. Furthermore, 20% found it difficult to balance the detox with work-related demands on digital tools, which limited their adherence. Despite these challenges, participants expressed increased mindfulness and a reduced dependency on devices by the end of the detox.

**Conclusion:** This study highlights the feasibility and potential benefits of a digital detox intervention among medics. The findings suggest that reducing device usage can significantly improve focus, stress levels, and work-life balance. Future studies should explore personalised and sustainable detox strategies that account for the unique demands of professional and personal digital use.

